# Asphaltene biodegradation and transcriptome responses of hydrocarbon degradation genes in soil by a novel tolerant fungal consortium

**DOI:** 10.1007/s11356-026-37580-8

**Published:** 2026-03-25

**Authors:** Mábel Sofía Barrera, Claudia Marín, Diana V. Cortés-Espinosa, German Zafra

**Affiliations:** 1 Grupo de Investigación en Bioquímica y Microbiología (GIBIM), Escuela de Microbiología, Universidad Industrial de Santander, Carrera 32 # 29-31, Bucaramanga, 680002 Santander Colombia; 2 Instituto Politécnico Nacional, Centro de Investigación en Biotecnología Aplicada, Carretera Estatal Tecuexcomac-Tepetitla, Km 1.5, Tlaxcala, 90700 México

**Keywords:** Fungal consortium, Asphaltene biodegradation, Microbial tolerance, Biodegradation genes, Expression profiling, Soil bioremediation

## Abstract

Soil contamination with petroleum asphaltenes represents a critical environmental hazard due to their recalcitrant nature and their potential long-term adverse effects on ecosystems, plants, animals, and human health. Bioremediation using microbial consortia capable of degrading heavy hydrocarbons offers a promising strategy for the efficient removal of asphaltenes from contaminated soils. This study aimed to develop a novel fungal consortium highly tolerant to asphaltenes for the bioremediation of contaminated soils and evaluate transcriptional responses during asphaltene degradation. The consortium, composed of *Aspergillus oryzae*, *Penicillium citrinum*, and *Aspergillus sydowii*, was evaluated in microcosm systems containing asphaltene-contaminated soil, and transcriptomic analyses were conducted to assess gene activity. Bioremediation assays revealed that the consortium not only removed but also mineralized asphaltenes, achieving a degradation of up to 55.6 wt% of 5000 mg of asphaltenes kg^−1^ in 30 days. Additionally, it exhibited remarkable tolerance to asphaltenes at concentrations exceeding 100,000 mg of asphaltenes l^−1^. Transcriptomic profiling indicated that cytochrome P450 monooxygenases and extradiol dioxygenases were primarily involved during the initial stages of degradation, followed by upregulation of genes encoding laccases and DyP-type peroxidases, suggesting a coordinated metabolic response to the breakdown of complex aromatic compounds. The consortium demonstrated the ability to adapt and overexpress hydrocarbon-degrading genes in response to asphaltene exposure, underscoring its potential as an effective and viable bioremediation tool.

## Introduction

Soil contamination with heavy petroleum fractions, such as asphaltenes, resins, and bituminous compounds, represents a significant environmental issue, primarily due to their high toxicity, low biodegradability, and long-term persistence, which adversely affect human and animal health. The presence of these compounds can alter the soil ecological balance, inhibit plant growth, and contaminate groundwater sources. Heavy petroleum fractions are generally resistant to microbial decomposition, leading to a prolonged accumulation of toxic hydrocarbons in contaminated soils. This ultimately alters their physicochemical composition, structure, and nutrient and oxygen levels, affecting their capacity to support living organisms (Brown et al. 2017). Asphaltenes are the heaviest fraction of crude oils. Due to their high molecular weight, complex and variable structure, including polyaromatic rings, the presence of alkyl chains and heavy metals, and high hydrophobicity, their resistance to microbial degradation is even greater than that of other heavy petroleum fractions (Hassanzadeh and Abdouss 2023). The remediation of asphaltene-contaminated soils presents considerable technical and economic challenges. Although conventional physical and chemical techniques can be employed to partially restore asphaltene-contaminated soils, the recalcitrant nature of asphaltenes limits the efficiency of their removal, reduces toxicity, and results in high costs for both implementation and maintenance of such approaches (Kshirsagar et al. 2020).

Bioremediation is an environmentally friendly and cost-effective biological approach that enables the microbial degradation of asphaltenes at high rates (Shahebrahimi et al. 2020). The use of degrading microbial consortia has been reported as an effective approach to the biodegradation of pure asphaltenes; however, most of these studies have been conducted using liquid cultures and non-contaminated soils (Shahebrahimi et al. 2020; Yanto and Tachibana 2014a). Recently, a highly tolerant bacterial consortium was reported to degrade up to 83 wt% of 10,000 mg of asphaltene kg^−1^ in soil after 7 weeks of treatment (Navas-Cáceres et al. 2023), demonstrating that microbial tolerance is a key factor in developing efficient and long-lasting degrading consortia for use in soil. In this context, filamentous fungi may exhibit adaptive advantages over bacteria in soils contaminated with heavy hydrocarbons. Besides representing an important part of soil biomass, fungi possess metabolic mechanisms that confer particular growth and adaptation characteristics, enabling them to survive and thrive in conditions of low water activity (Vaksmaa et al. 2023). Several fungal species can survive and succeed in soils severely polluted with heavy hydrocarbons due to their ability to degrade long-chain or multi-ring hydrocarbons (Dinakarkumar et al. 2024). The ability of fungi to degrade xenobiotics and heavy hydrocarbons may be significantly influenced by the presence and expression of hydrocarbon degradation genes, including oxidative and ligninolytic enzymes such as laccases, peroxidases, cellulases, and oxygenases (Benguenab and Chibani 2021). The presence and production of these enzymes depend on fungal genotypes and biotic and abiotic factors that strongly influence survival and degradation rates in polluted soils (Ningombam et al. 2025). A highly tolerant and biologically compatible fungal consortium would exhibit a high bioremediation potential in asphaltene-contaminated soils, which could offer advantages over bacterial consortia. Hence, this work aimed to (1) develop a novel fungal consortium highly tolerant to asphaltenes for the bioremediation of asphaltene-contaminated soils, (2) evaluate the asphaltene degradation efficiency of the consortium under different soil conditions, and (3) assess changes in the expression of fungal oxidoreductases and ligninolytic genes during the bioremediation of asphaltene-contaminated soils. This approach provides new insights into the mechanisms employed by fungi to tolerate and degrade highly recalcitrant pollutants such as asphaltenes and identifies the potential of the fungal consortium as a tool for soil bioremediation.

## Materials and methods

### Materials

The solvents used for the preparation and extraction of the soil hydrocarbons (methanol and toluene) were of analytical grade and were provided by Sigma-Aldrich. Samples of heavy crude oil (API gravity of 17.8°; fraction composition of 38 wt% saturates,10 wt% aromatics, 21 wt% resins, 11 wt% asphaltenes) and pure asphaltenes (molecular composition of 84 wt% carbon, 8 wt% hydrogen, 1.5 wt% nitrogen, 4.5 wt% sulfur, 1.5 wt% oxygen, 406 mg kg^−1^ Ni, 1601 mg kg^−1^ V; MW = 2000 Da) were provided by the Colombian Petroleum Institute (Colombia). The Czapek medium used for the isolation of the microorganisms contained 3 g l^−1^ NaNO_3_, 1 g l^−1^ K_2_HPO_4_, 0.5 g l^−1^ MgSO_4_, 0.5 g l^−1^ KCl, 0.01 g l^−1^ FeSO_4_, and 15 g l^−1^ bacteriological agar (Sigma-Aldrich). The potato dextrose (PDA) medium used contained 4 g l^−1^ potato extract, 20 g l^−1^ dextrose, and 15 g l^−1^ bacteriological agar (Sigma-Aldrich). Yeast extract peptone glucose (YPG) liquid medium contained 20 g l^−1^ glucose, 30 g l^−1^ peptone, and 10 g l^−1^ yeast extract. The raw sugarcane bagasse used in the hydrocarbon biodegradation tests contained 31.98 wt% carbon, 0.27 wt% nitrogen, and 0.007 wt% phosphorus.

### Isolation and identification of asphaltene‑degrading fungi from soil

Samples of aged heavy crude oil–contaminated soil were obtained from the Lisama oil field in Santander, Colombia (7°05′39.7″N; 73°33′29.7″W). Composite samples of 100 g each were collected randomly at a depth of 30 cm, following the procedures described by the U.S. EPA (Simmons 2023). Uncontaminated soil samples were collected from the same coordinates. The samples were stored at 4 °C until processed. Native filamentous fungi were isolated by diluting 1 g of soil in 9 ml of liquid Czapek medium supplemented with 0.1 wt% heavy crude oil (API gravity of 17.8°) as the sole carbon source. Cultures were incubated at 30 °C for 48 h under constant shaking at 200 × g. Subsequently, 100 µl of the culture supernatants was inoculated into plates of solid Czapek medium supplemented with 0.1 wt% heavy crude oil, aiming to select isolates that only use this compound as the sole source of carbon. The plates were incubated at 30 °C until visible growth was observed. After sporulation, colonies were picked and transferred to plates containing PDA media.

Native fungal isolates were identified by genomic DNA extraction followed by polymerase chain reaction (PCR) amplification and sequencing of partial ribosomal DNA. Genomic DNA from each pure isolate was obtained from fresh mycelia produced in the YPG liquid media through the DNeasy PowerSoil Pro Kit (QIAGEN). Measurements to confirm the DNA concentration and quality were performed on an NP80 NanoPhotometer (IMPLEN). PCR amplification of the Internal Transcribed Spacer (ITS) regions (ITS1 and ITS2) and the 5.8S ribosomal RNA gene was performed with the primers ITS5 (5′-GGAAGTAAAAGTCGTAACAAGG-3′) and ITS4 (5′-TCCTCCGCTTATTGATATGC-3′) (White et al. 1990). The reaction conditions consisted of 10 ng of genomic DNA, 1 µM of each primer, 2 mM MgCl_2_, a 200 µM dNTP mixture, and 1 U of Taq DNA polymerase (Thermo Scientific) in a final volume of 20 µl. The amplification conditions consisted of 33 cycles of 95 °C for 30 s, 49 °C for 45 s, and 72 °C for 45 s, with a final extension step of 72 °C for 7 min. The PCR products were analyzed by agarose gel electrophoresis, purified using the Wizard SV Gel and PCR Clean-Up System (Promega), and sequenced in a 3730xl DNA analyzer (Applied Biosystems), with the oligonucleotide ITS5 as sequencing primer. The resulting sequences were quality-checked using Bioedit v7.2.5 (Hall, 1999) and compared with the GenBank database using the web-based basic local alignment tool (nucleotide BLAST). Ribosomal sequences were deposited in the NCBI GenBank database (https://www.ncbi.nlm.nih.gov/genbank/). Phylogenetic analysis of the sequences was performed using the neighbor-joining algorithm with the Kimura two-parameter model using MEGA 11 software (Tamura et al. 2021).

### Development of an asphaltene-degrading and highly tolerant fungal consortium

The development of the asphaltene-degrading fungal consortium was based on three main characteristics: (1) the growth capacity of the fungal isolates using heavy crude oil as the only carbon source; (2) the tolerance of the fungal isolates to high concentrations of pure asphaltenes; and (3) the absence of observable antagonistic relationships between isolates. The tolerance of fungal isolates to several doses of pure asphaltenes was tested using surface plate assays, supplementing PDA plates with 1000, 10,000, 30,000, 60,000, 80,000, and 100,000 mg of asphaltenes l^−1^ previously dissolved in analytical grade toluene, as described by Zafra et al. (2017). The plates were inoculated in the center with 1 × 10^5^ spores of each individual isolate and incubated for 10 days at 25 °C. Control plates without asphaltenes and inoculated with each of the isolates were included. Isolates showing visible mycelial growth after incubation were considered tolerant to the corresponding dose of asphaltenes in the medium. In vitro evaluation of fungal antagonism was carried out using dual culture plate assays between the most asphaltene-tolerant isolates. Single PDA medium plates were inoculated with 1 × 10^5^ fresh spores of two fungal isolates, each separated from the other by 4 cm and incubated at 25 °C until inhibition halos or antagonistic effects between colonies were observed. The growth rates of individual fungal isolates were determined by inoculating 1 × 10^4^ spores of each of the isolates on PDA plates supplemented with 1000, 10,000, 30,000, or 100,000 mg of asphaltenes l^−1^, measured daily for 10 days, and reported as millimeters of colony expansion per day.

### Evaluation of asphaltene degradation in soil by the fungal consortium

The ability of the fungal consortium to degrade asphaltenes in contaminated soils was evaluated in 100-ml glass reactors using a combination of bioaugmentation (by inoculating the fungal consortium) and biostimulation (by providing sugarcane bagasse as a soil texturizer). The biodegradation conditions were set as follows: Treatment T1 (bioaugmentation with the consortium) consisted of reactors containing 6.65 g of sterile (autoclaved) soil spiked with 5000 mg of asphaltenes kg^−1^, and 0.35 g of sterile sugarcane bagasse and inoculated with 2 × 10^6^ spores of each of the isolates selected to be part of the consortium. This treatment was designed to evaluate the ability of the consortium alone to degrade asphaltenes in the soil (without the native soil microbiota). Treatment T2 (bioaugmentation with the consortium + biostimulation of the native soil microbiota) consisted of reactors containing 6.65 g of non-sterile soil (not autoclaved) spiked with 5000 mg of asphaltenes kg^−1^, 0.35 g of sterile sugarcane bagasse, and inoculated with 2 × 10^6^ spores of each of the isolates selected to be part of the consortium. This treatment was designed to evaluate the combined ability of the consortium and the native soil microbiota to degrade asphaltenes in the soil. Treatment T3 (biostimulation of the native soil microbiota) consisted of reactors containing 6.65 g of non-sterile soil (not autoclaved) spiked with 5000 mg of asphaltenes kg^−1^, 0.35 g of sterile sugarcane bagasse and not inoculated with the fungal consortium. This treatment was used to evaluate the ability of the native soil microbiota alone to degrade asphaltenes in the absence of the consortium. Control reactors consisting of uncontaminated (without asphaltenes) and non-sterile (not autoclaved) soils inoculated with the consortium were included to determine respiratory levels of the soil in the absence of asphaltenes. Additionally, abiotic controls consisting of sterile reactors containing uninoculated autoclaved soil spiked with asphaltenes were included to compensate for asphaltene adsorption losses. All reactors were incubated for 30 days at 25 °C and aerated every 48 h with sterile air to maintain aerobic conditions. All assays were performed in triplicate.

The survival of the fungal isolates at the end of the degradation process was determined through low-stringency single-specific primer PCR (LSSP-PCR). Genomic DNA was extracted from the soil samples using the DNeasy PowerSoil Pro Kit (QIAGEN). LSSP-PCR reactions were carried out by purifying ITS1-2 amplicons obtained from soil with the primers ITS4/ITS5 and performing a second round of amplification using only the ITS4 primer. Reactions were carried out in a final volume of 10 µl containing 2 U of Taq DNA polymerase (Invitrogen), 200 µM dNTPs, 3 mM MgCl2, and 5 µM of primer P27F. The amplification conditions were as follows: initial denaturation at 95 °C for 5 min, followed by 40 cycles at 94 °C for 30 s, 32 °C for 30 s, and 72 °C for 30 s. The resulting products were resolved on 3 wt% agarose gels and the resulting band profiles analyzed with PyElph v.1.4 (Pavel and Vasile 2012).

### Heterotrophic activity and analytical monitoring during biodegradation

Microbial respiratory activity during soil bioremediation was determined by measuring the amount of carbon dioxide (CO_2_) released every 48 h with a 7752 AZ CO_2_ detector (AZ Instruments Corp.) equipped with a nondispersive infrared sensor and expressed as mg CO_2_ released per g of dry matter (soil plus sugarcane bagasse). Asphaltene degradation in the soil was quantified using the shaking-centrifugation method (ASTM 2022), with a mixture of methanol and toluene used as the extracting solvent in a 2:1 ratio. The resulting organic extracts were evaporated and concentrated to determine the concentration of residual asphaltenes in the soil by gravimetry, according to the ASTM D6560 standard method (ASTM 2022). All assays were performed in triplicate.

### Evaluation of the expression of hydrocarbon oxidative and ligninolytic genes during asphaltene biodegradation by the fungal consortium

Relative changes in the expression of fungal genes involved in asphaltene degradation during the bioremediation of contaminated soils were investigated by RT-qPCR. Total RNA was obtained from treatment T1 (asphaltene-contaminated soils inoculated with the fungal consortium) and control soils (uncontaminated soils inoculated with the fungal consortium) on days 1, 15, and 30 of degradation, using the Soil Total RNA Purification Kit (Norgen Biotek, CA, USA). Before extraction, the soil samples were washed twice with RNAlater™ solution (Invitrogen) to help wash humic acids and stabilize RNA. RNA integrity was estimated by the absorbance ratio of 260/280 nm on an NP80 NanoPhotometer (IMPLEN) and on 1 wt% agarose gels. cDNA was synthesized from total RNA with the Verso cDNA Synthesis Kit (Thermo Scientific) according to the manufacturer's instructions.

Specific PCR primers were designed to measure the relative gene expression levels of seven genes encoding oxidative fungal enzymes involved in lignocellulose, aromatic hydrocarbon, and xenobiotic degradation, including laccases, mono and dioxygenases, and peroxidases, in *Aspergillus* and *Penicillium* (Table 1). The expression levels of the beta-tubulin (Tamano et al. 2013) and H3 histone (Li et al. 2017) genes were used as a reference to normalize gene expression levels. RT-qPCR experiments were performed using a CFX96 thermal cycler (Bio-Rad Laboratories) and the resulting data were analyzed using the 2^−∆∆Ct^ method (Livak and Schmittgen 2001) implemented in CFX Maestro ™ software (Bio-Rad Laboratories). The reactions were set at a final volume of 20 µl and contained a 2X iTaq Universal SYBR® Green One-Step reaction mix (Bio-Rad), 1 µM forward and reverse primers, and 1 µl of cDNA. The amplification conditions were as follows: 5 min at 95 °C, 40 cycles of 10 s at 95 °C and 10 s at 60 °C, and a final melting curve with increments of 0.5 °C every 5 s in a linear gradient of 60 to 90 °C. Reactions were performed in triplicate and no-template controls were included for each reaction. The amplicon sizes were verified on agarose gels.

**Table 1:** Primers used for the evaluation of fungal hydrocarbon-degrading gene expression by RT-qPCR

Organism	Gene	Primer name and sequence (5′−3′)	Amplicon length (bp)	Reference
*Aspergillus oryzae*	Extradiol ring-cleavage dioxygenase, class III enzyme, subunit B (*ligB*)	LigB-AoF: GGCTCTGACGGATTTGTTGA	179	This study
		LigB-AoR: TGTTGGCTACCTGACCGAAT		
	Cytochrome P450 monooxygenase (*cyp*)	cyp-AoF: CTGCAAAACGATCGCTGAGA	200	This study
		cyp-AoR: CAGTCATCCTTTTCTGGGCG		
	Blue-copper laccase (*tilA*)	tilA-AoF: GTTGGCGTGCTTGTGAATTG	190	This study
		tilA-AoR: CTCCTACACCTGGACCTTGG		
	Chloroperoxidase (*cpo*)	Clp-AoF: TCTACTTCGGCGACAACCAT	156	This study
		Clp-AoR: CGGAGAGGTCAAATGTTGGG		
	Beta tubulin (*tub*)	TubF: GAAACTCCACCTCCATCCA	112	Tamano et al. (2013)
		TubR: ATCTCGTCCATACCCTCACC		
*Penicillium* *citrinum*	Extradiol ring-cleavage dioxygenase, class III enzyme, subunit B (*ligB*)	LigB-PcF: ATTATTGGGACTGGAGGGGC	185	This study
		LigB-PcR: AACCTCGTCATAGCTCTCCG		
	Cytochrome P450 monooxygenase (*cyp*)	Cyp-PcF: TACTTCCTCTTGCTGGCCTT	154	This study
		Cyp-PcR: GGACCGTAAATTCGCGACC		
	DyP-type peroxidase (*dyp*)	Dyp-PcF: CAAAACGATCGAGGCCTCAT	179	This study
		Dyp-PcR: TACTCGGTCTTGTCTGCTGG		
	H3 histone (H3)	H3F: TCCGTGAGATCCGTCGCTACCA	141	Li et al. (2017)
		H3R: ACTCCTGAAGAGCACCGATGGC		

### Statistical analysis

Data from CO_2_ measurements and asphaltene degradation in soils were analyzed using an analysis of variance (ANOVA) and Tukey’s multiple comparison test using the software GraphPad Prism v.9.4.1, with the *p*-values < 0.05 considered statistically significant.

## Results

### Native asphaltene‑degrading fungi isolated from contaminated soil

**Fig. 1:**
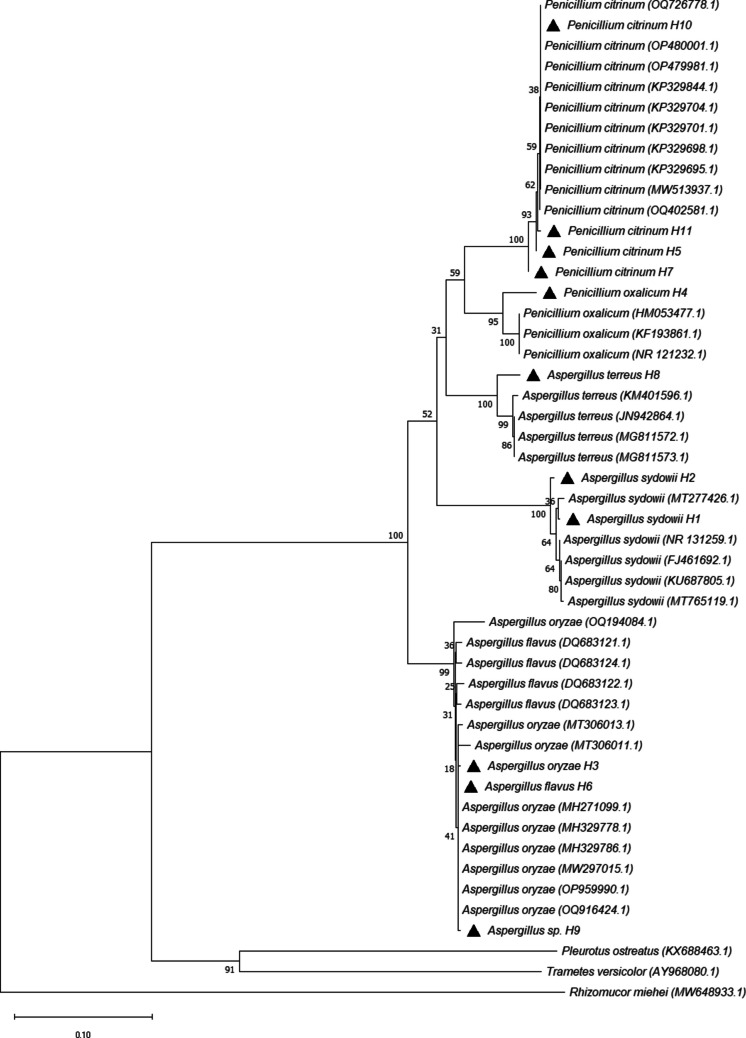
Neighbor-joining tree showing the clustering and similarities of fungal isolates based on ITS1, 5.8S ribosomal RNA, and ITS2 gene sequences. The numbers on the nodes denote percentage bootstrap values based on 1000 replicates. The black triangles indicate the sequences corresponding to the organisms isolated in this study. The GenBank accession numbers of the reference sequences are indicated in parentheses

A total of 11 native fungal isolates were obtained from aged, heavy crude oil–contaminated soil, all of which exhibited tolerance to crude oil and the capacity to use it as a carbon and energy source. The morphological analysis of the isolates revealed similarities in their microscopic and macroscopic morphologies, showing typical characteristics of the genera *Penicillium* and *Aspergillus*. As per the above findings, the analysis of the ITS1, 5.8S ribosomal RNA, and ITS2 fungal sequences indicated that the isolates corresponded to seven different species of the *Penicillium* and *Aspergillus* genera (Table 2) and clustered into five clades (Fig. 1). The isolates were phylogenetically diverse, and even those identified as belonging to the same species (e.g., *Aspergillus sydowii* H1 and H2; *Penicillium citrinum* H5, H7, and H10) were found not to be 100% similar. The resulting DNA sequences were deposited in GenBank (Table 2).

**Table 2:** BLAST identification of fungal isolates obtained from heavy crude oil-contaminated soils based on ribosomal ITS sequences

Isolate	Taxon group (species)	NCBI accession^a^	Identity (%)
H1	*Aspergillus sydowii*	PQ012221.1	99.26%
H2	*Aspergillus sydowii*	PQ012222.1	99.03%
H3	*Aspergillus oryzae*	PQ012223.1	99.81%
H4	*Penicillium oxalicum*	PQ012224.1	98.9%
H5	*Penicillium citrinum*	PQ012225.1	99.42%
H6	*Aspergillus flavus*	PQ012226.1	99.03%
H7	*Penicillium citrinum*	PQ012227.1	99.04%
H8	*Aspergillus terreus*	PQ012228.1	98.40%
H9	*Aspergillus* sp.	PQ012229.1	99.82%
H10	*Penicillium citrinum*	PQ012230.1	100%
H11	*Penicillium citrinum*	PQ012231.1	99.42%

^a^Reported accession number in the NCBI GenBank database

### Development of a highly tolerant and asphaltene-degrading fungal consortium

**Fig. 2:**
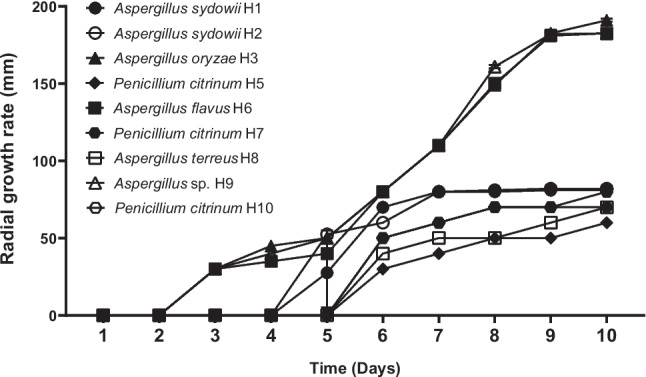
Radial growth rates of individual fungal strains cultivated on PDA plates supplemented with 100,000 mg of asphaltenes l^−1^ after 10 days of growth

**Fig. 3:**
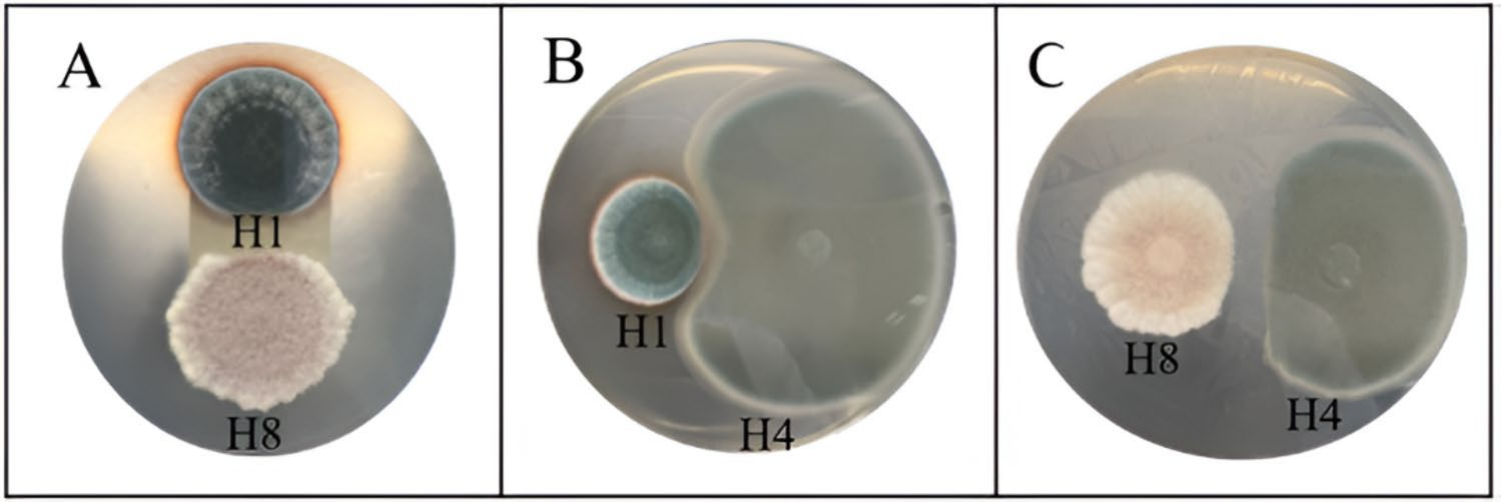
Representative dual culture plate assays showing antagonism between fungal isolates on PDA plates after ten days of growth. **A** H8 (*Aspergillus terreus*) vs. H1 (*Aspergillus sydowii*); **B** H1 (*Aspergillus sydowii*) vs. H4 (*Penicillium oxalicum*); **C** H8 (*Aspergillus terreus*) vs. H4 (*Penicillium oxalicum)*

Fungal isolates presented visible growth after 10 days on PDA plates supplemented with 1000, 10,000, and 30,000 mg of asphaltenes l^−1^, and nine of them (isolates H1, H2, H3, H5, H6, H7, H8, H9, and H10) were able to grow at a concentration of 100,000 mg of asphaltenes l^−1^, with variations in their radial growth rates and sporulation patterns as the asphaltene concentration increased. These nine tolerant isolates were used for further testing to develop the fungal consortium, and three of them, *Aspergillus oryzae* H3, *Aspergillus flavus* H6, and *Aspergillus* sp. H9, exhibited the highest radial growth rates in all asphaltene concentrations tested, particularly at 100,000 mg l^−1^ (Fig. 2). Among the isolates tolerant to 100,000 mg of asphaltenes l^−1^, the main antagonist to the other isolates in the dual culture assays was isolate H8 (*Aspergillus terreus*) (Fig. 3). Isolates H1 (*Aspergillus sydowii*), H3 (*Aspergillus oryzae*), H4 (*Penicillium oxalicum*), H6 (*Aspergillus flavus*), H9 (*Aspergillus* sp.), H5, and H10 (*Penicillium citrinum*) did not produce or suffer any antagonistic effects. Based on tolerance level and radial growth rate in the presence of asphaltenes, as well as the lack of antagonism, the isolates selected to be part of the fungal consortium were *Aspergillus sydowii* (H1), *Aspergillus oryzae* (H3), and *Penicillium citrinum* (H10). These were preserved in the microbial culture collection “Cepario LMMA-UIS,” registered in the Colombian National Registry of Biological Collections (RNC Registry No. 241).

### Asphaltene biodegradation in soil by the fungal consortium

**Fig. 4:**
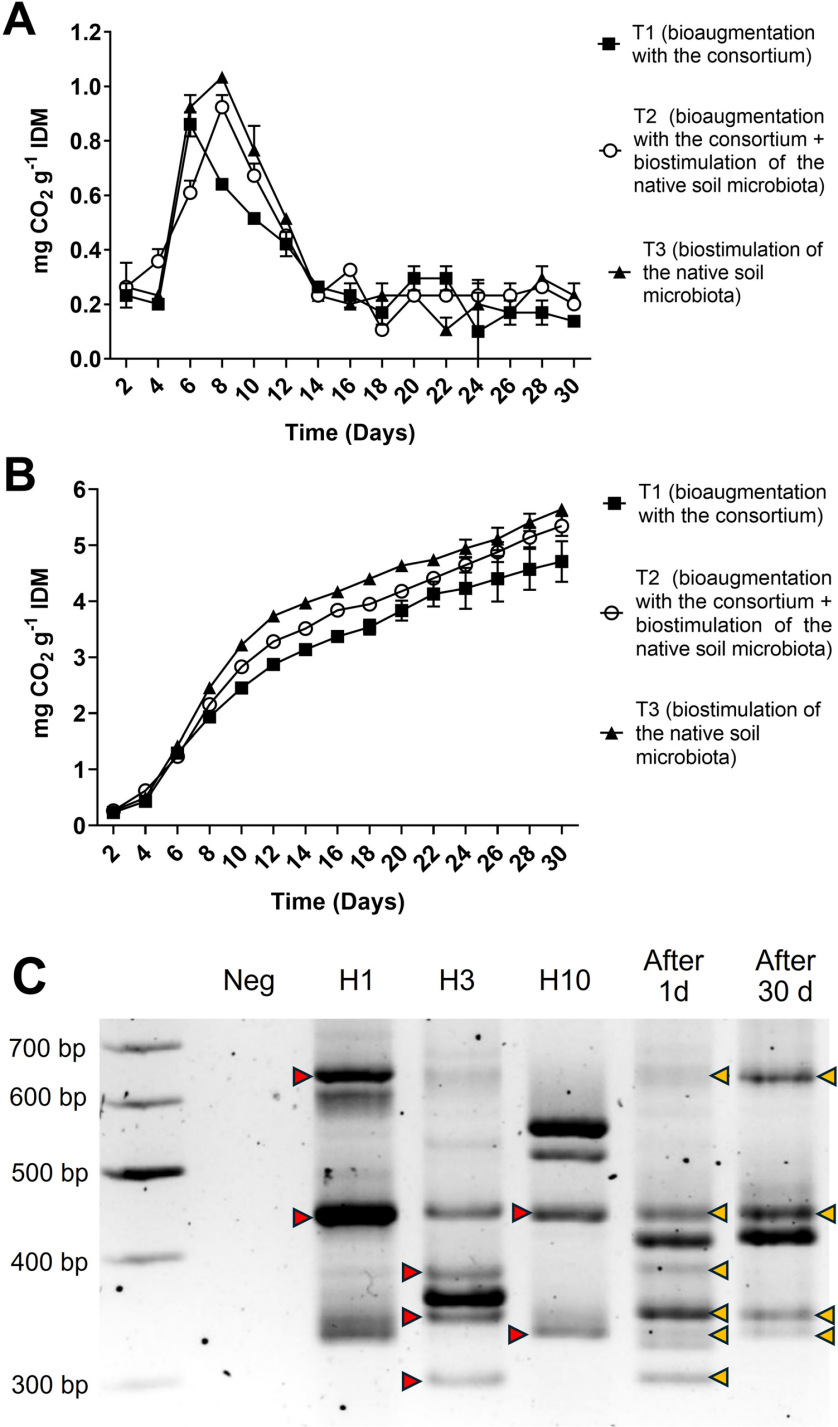
Instantaneous (**A**) and accumulated (**B**) CO_2_ production during bioremediation of soils contaminated with 5000 mg of asphaltenes kg^−1^ and inoculated with the fungal consortium for 30 days. The error bars represent the standard errors of the means (*n* = 3). IDM, initial dry matter. **C** LSSP-PCR profiles from individual fungal isolates and soil contaminated with 5000 mg of asphaltenes kg^−1^ and inoculated with a combination of isolates H1, H3, and H10. Yellow arrows indicate the individual bands that remained after 1 and 30 days of treatment, which, together with microbial isolation in PDA medium, confirmed the survival of all fungal isolates in contaminated soils. Neg, negative control

**Fig. 5:**
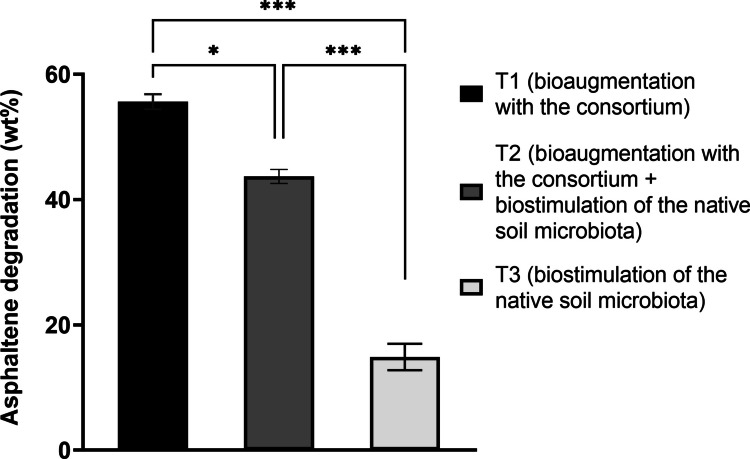
Asphaltene degradation in soils contaminated with 5000 mg of asphaltenes kg^−1^ after a 30-day treatment under different bioremediation conditions. The degradation efficiencies differed significantly among the three treatments (T1, T2, and T3). The error bars represent the standard errors of the means (*n* = 3). *p = 0.01. **p = 0.0007. ***p = 0.0002

Asphaltene biodegradation by the fungal consortium was evaluated in soils contaminated with asphaltenes, and constant microbial respiratory activity was detected under all treatment conditions throughout the course of the biodegradation experiments, indicating sustained microbial mineralization of soil compounds and/or asphaltenes. Generally, there was a noticeable increase in the amount of CO_2_ produced during the first week of all treatments, followed by a rapid decrease until day 14 and low but sustained CO_2_ production until day 30 (Fig. 4A). Soils subjected to treatment T2 (bioaugmentation with the consortium combined with biostimulation of the native soil microbiota) produced higher levels of CO_2_ than those of treatment T1 (bioaugmentation with the consortium alone), although the difference was not statistically significant (*p* = 0.1417) (Fig. 4B). Despite this, there was a significantly higher asphaltene degradation efficiency in contaminated soils containing only the fungal consortium (treatment T1) (55.62%) than in soils containing the native microbiota plus the fungal consortium (treatment T2) (43.70%) (*p* = 0.0091) (Fig. 5). Compared to the addition of the consortium, the native soil microbiota (treatment T3) achieved only 14.89% degradation of asphaltenes after 30 days. LSSP-PCR profiles of soils and isolation on PDA on day 30 confirmed that the three isolates comprising the fungal consortium were present throughout the 30 days of degradation (Fig. 4C).

### Expression of hydrocarbon degradation genes during asphaltene biodegradation by the fungal consortium

**Fig. 6:**
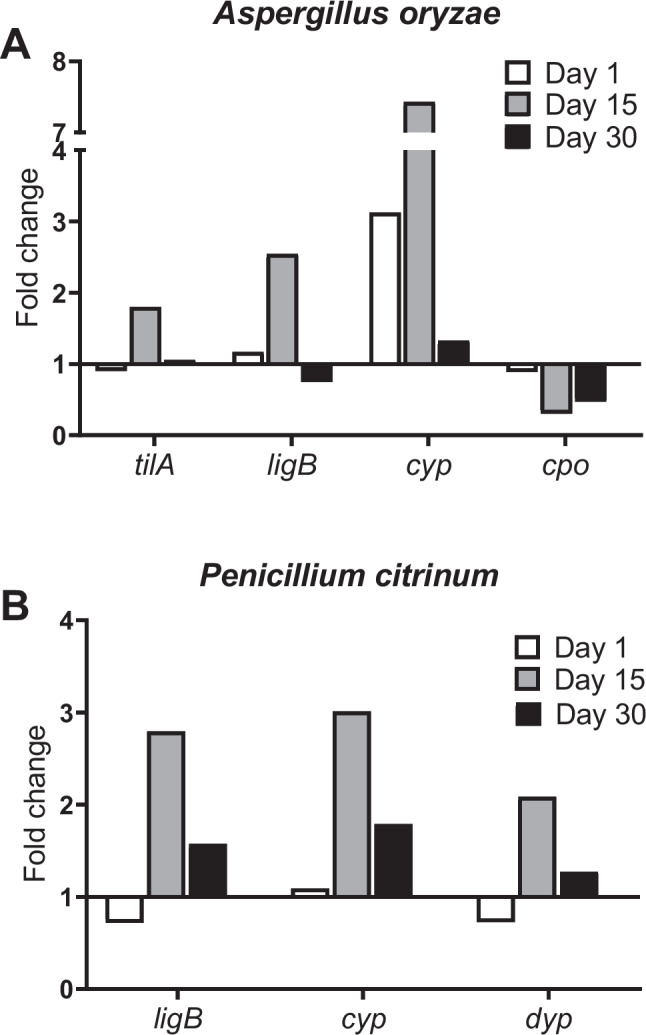
Changes in relative expression levels of fungal blue-copper laccase (*tilA*), DyP-type peroxidase (*dyp*), chloroperoxidase (*cpo*), cytochrome P450 monooxygenase (*cyp*), and extradiol ring-cleavage dioxygenase *(ligB*) genes during bioremediation of soils contaminated with 5000 mg of asphaltenes kg^−1^and inoculated with the fungal consortium for 30 days (treatment T1). The controls consisted of uncontaminated soils (non-spiked with asphaltenes) inoculated with the consortium. **A** *Aspergillus oryzae* genes; **B** *Penicillium citrinum* genes

Although the expression of all evaluated hydrocarbon degradation genes was detected throughout the bioremediation treatment, the presence of asphaltenes in the soil induced significant increases in the expression of specific genes at various stages of biodegradation. For example, *A. oryzae* cytochrome P450 (*cyp*) genes were overexpressed at early (day 1) and intermediate stages (day 15) of biodegradation, with the greatest detected fold change (7.5) compared to the uncontaminated control (Fig. 6A), while *P. citrinum cyp* genes overexpressed only from day 15 to day 30 (Fig. 6B). The expression patterns of extradiol dioxygenase genes (*ligB*) in response to asphaltenes were consistent with those of *cyp* genes, being first overexpressed by *A. oryzae* (day 15) and later by *P. citrinum* (days 15 and 30). On the other hand, the expression of ligninolytic genes encoding extracellular laccase (*tilA*) and DyP-type peroxidase (*dyp*) was constant during the 30 days of treatment but was overexpressed only on day 15. Generally, day 15 of degradation was the time point at which more genes were overexpressed and with higher fold change values for both *A. oryzae* and *P. citrinum*. Conversely, the expression of the chloroperoxidase-encoding gene (*cpo*) was downregulated in the presence of asphaltenes. The transcriptome responses of *Aspergillus sydowii* genes were not evaluated since there were no sequences of the *cyp*, *ligB*, *dyp*, *tilA*, or *cpo* genes available for primer design in the NCBI databases or other sequence repositories at the time of evaluation.

## Discussion

Fungi constitute most of the soil microbial biomass and are known for their ability to degrade recalcitrant compounds due to their metabolic versatility and ability to colonize different types of habitats, producing low-specificity enzymes that contribute to the degradation of environmental contaminants (Harms et al. 2011). In recent years, filamentous fungi have been recognized as efficient hydrocarbon degraders, particularly species belonging to the *Aspergillus, Penicillium, Fusarium, Trichoderma, Paecilomyces,* and *Talaromyces* genera, among others (Essabri et al. 2019). Although several studies have demonstrated the ability of ascomycetes to metabolize hydrocarbons, few have investigated the fungal biodegradation efficiency of asphaltenes in contaminated soils (Ramoutar et al. 2019). This study aimed to isolate native fungal isolates with extreme tolerance levels to asphaltenes to develop a novel fungal consortium capable of efficiently degrading asphaltenes in highly contaminated soils. Eleven native fungal isolates, able to tolerate and use heavy crude oil as a source of carbon and energy, were isolated from aged, heavy crude oil–contaminated soil. Although several of the isolated fungal species have already been described as hydrocarbon-degrading microbes, such as *Penicillium citrinum* (Barnes et al. 2018)*, Aspergillus flavus* (Zafra et al. 2014), *Aspergillus sydowii* (Khandelwal et al. 2023), and *Aspergillus oryzae* (Asemoloye et al. 2020; Barnes et al. 2018), none of the isolated species has been reported to partially or totally degrade pure asphaltenes. This study represents a pioneering effort to evaluate fungal tolerance to extreme asphaltene concentrations and assess microbial antagonism among degrading strains, resulting in a robust, non-antagonistic consortium for efficient in-soil asphaltene biodegradation.

Proper selection of efficient microorganisms for soil bioremediation requires characterization of their tolerance to toxic contaminants, as the lack of tolerance of microbial isolates is a key factor that contributes to the long-term persistence of environmental contaminants through inhibition of metabolism and cell growth (Mahmud et al. 2022). Various fungal species have shown high tolerance to concentrations of up to 50 wt% of heavy crude oil (Cáceres-Zambrano et al. 2024) and up to 30,000 mg of asphaltenes l^−1^ (Yanto and Tachibana 2014b), while several bacterial degrading species have shown tolerance levels of up to 60,000 mg of asphaltenes l^−1^ (Navas-Cáceres et al. 2023). However, only few studies have examined how fungal tolerance to heavy hydrocarbons relates to improved bioremediation performance in soil, leaving an important knowledge gap regarding the mechanistic links between tolerance traits and contaminant removal efficiency (Al‑Zaban et al. 2021; Fallahi et al. 2023). The fungal isolates evaluated in this study presented higher tolerance levels than those already reported, even at concentrations as high as 100,000 mg of asphaltenes l^−1^, especially those identified as *Aspergillus oryzae* and *Aspergillus flavus.* These species have been described as tolerant to significant amounts of polycyclic aromatic hydrocarbons (PAHs) and have been successfully used in the degradation of PAHs and total petroleum hydrocarbons in vitro (Asemoloye et al. 2020). The high tolerance found in the studied isolates is consistent with previous research showing that prolonged exposure to hydrocarbons selects microbial populations with increased tolerance and degradative capacity (Al-Zaban et al. 2021), positively influencing their survival in contaminated soils (Zafra et al. 2014). Specifically, the three species included in the degrading consortium (*Aspergillus oryzae*, *Aspergillus sydowii*, and *Penicillium citrinum*) have been studied for their ability to degrade several toxic compounds, but not for the biodegradation of asphaltenes. For instance, Kumar et al. (2023) reported that an isolate of *P. citrinum* had a high emulsifying activity of petroleum hydrocarbons, which are critical components in the breakdown of hydrocarbons. Asemoloye et al. (2020) reported that *A. oryzae* produces a variety of enzymes related to high degradative activity against all hydrocarbon components of motor oil. Conversely, *Aspergillus sydowii* has been isolated from weathered oil tarballs, although it has shown a lower degradation efficiency than *P. citrinum* in the degradation of long hydrocarbon chains (Barnes et al. 2018). These tolerance capabilities and the absence of antagonistic interactions enable the use of the fungal consortium in bioremediation strategies, enabling synergistic or co-metabolic processes during the degradation of the contaminant in soil (Zhu et al. 2025).

The inoculation of the fungal consortium led to constant CO_2_ production during the course of the biodegradation experiments in asphaltene-contaminated soils, indicative of sustained microbial mineralization activity. The significant increase in CO_2_ concentrations during the first week of all treatments suggests an efficient adaptation of the consortium to contaminated soils, as well as the use of low-complexity carbon sources present in the soil, sugarcane bagasse, and light fractions derived from asphaltenes. These results are consistent with a report showing efficient and constant mineralization of asphaltenes in soil by a bacterial consortium with characteristics similar to those developed here, but with considerably lower tolerance levels (Navas-Cáceres et al. 2023). The maximum asphaltene degradation efficiency (55.62 wt%) was obtained when the consortium was inoculated in the absence of native microbiota, compared to soils containing native microbiota and the fungal consortium (43.70 wt%) or only native microbiota (14.89%). This indicates that the consortium is highly effective at degrading asphaltenes on its own, unaided by soil native microorganisms, which could be a potential advantage for the treatment of contaminated soils with low microbial counts or with a native microbiota strongly inhibited due to asphaltene toxicity. Previous studies have shown that native soil microbiota alone can accelerate the degradation of low concentrations (300 to 1400 mg kg^−1^) of petroleum aliphatic fractions (Guirado et al. 2023) and polycyclic aromatic hydrocarbons mixtures (Fallahi et al. 2023) present in soil but do not degrade asphaltene fractions, especially in soils with a higher content of contaminants (Galitskaya et al. 2021). Even when the native microbiota was present, the fungal consortium contributed to a significant increase in asphaltene degradation efficiency. The ability to mineralize asphaltenes with or without native microbiota suggests that the consortium could adapt to various soil conditions and degrade synergistically in both cases.

Compared to other studies that evaluated the fungal degradation of asphaltenes in soils (Yanto and Hidayat 2020; Yanto and Tachibana 2014b), the fungal consortium described here was challenged with greater amounts of asphaltenes (5000 mg kg^−1^) and achieved notably high biodegradation efficiencies (55.62 wt%). Together, these results show that the addition of the developed fungal consortium significantly improved asphaltene biodegradation compared to the native soil microbiota and indicates that the high CO_2_ rates produced are associated with the mineralization of asphaltenes in soil. LSSP-PCR profiles and soil microbial isolation confirmed that the three isolates comprising the fungal consortium survived throughout the 30 days of degradation, which was consistent with the high levels of tolerance to asphaltenes that improved their adaptability. To the best of our knowledge, based on publicly available information, this is the first report of *Aspergillus sydowii*, *Aspergillus oryzae*, and *Penicillium citrinum* being able to tolerate and mineralize high amounts of asphaltenes in soil.

Fungi produce a wide range of cell-bound, intracellular, and extracellular enzymes (e.g., oxidoreductases) primarily intended to break down complex lignocellulosic compounds, which are also involved in the degradation by co-metabolism of many other environmental contaminants (Al-Zaban et al. 2021; Baker et al. 2019). The activities of the main ligninolytic enzymes such as laccases and peroxidases are increased and complemented by other enzymes involved in the degradation of aliphatic and aromatic compounds, such as lipases and mono- and dioxygenases (Vaksmaa et al. 2023). Thus, studying the expression of these hydrocarbon degradation genes is crucial for understanding how the fungi involved in asphaltene biodegradation interact, transform, and mineralize the hydrocarbon complex compounds, as the lignin structure resembles that of the asphaltene fraction with multiple rings joined by alkyl chains (Zhang et al. 2021). We monitored the expression of seven of these genes in two of the three species included in the degrading consortium (*Aspergillus oryzae* and *Penicillium citrinum*) during the degradation of asphaltenes in the soil.

The presence of asphaltenes in soil significantly increased the expression of certain genes at specific stages of biodegradation. Genes encoding cytochrome P450 (*cyp*), a family of monooxygenases involved in fungal adaptability, organic matter degradation, secondary metabolite biosynthesis, and ecological functions (Khan and Murphy 2022), appeared to be major mediators involved in asphaltene degradation, first overexpressed by *A. oryzae* and then by *P. citrinum*. Cytochrome P450 enzymes are involved in the breakdown of xenobiotics and organic pollutants, as well as the O-demethylation of lignin, which can produce key intermediate molecules leading to mineralization (Harlington et al. 2022). Pure lignin induces the overexpression of genes encoding cytochrome P450 in white-rot fungi as a crucial step in the breakdown of lignin and probably also plays an essential role in the breakdown of asphaltenes in yeast (Buarque et al. 2025), as do hydroxylating-ring dioxygenases (Ribeiro Tomé et al. 2024). Transcriptomic and enzymatic studies of hydrocarbon‑adapted fungi have shown that inducible isoforms of P450 enzymes are commonly upregulated after exposure to complex petroleum fractions and substrates similar to lignin (Dinakarkumar et al. 2024), supporting the interpretation that these enzymes initiate asphaltene degradation in our experiments. The expression response of the *ligB* genes to asphaltenes was similar to that of the cytochrome *cyp* genes, in consonance with reports of the simultaneous involvement of mono- and dioxygenases in complex aromatic and lignin degradation (Kumar and Chandra 2020) and the upregulation of genes encoding dioxygenases during fungal lignin degradation (Ribeiro Tomé et al. 2024), further confirming that these enzymes are critical for the breakdown of complex aromatic compounds such as asphaltenes in contaminated soils. The expression of the ligninolytic genes encoding extracellular laccase (*tilA*) and DyP-type peroxidase (*dyp*), high redox potential broad-spectrum fungal enzymes used to degrade complex polyaromatic and xenobiotic compounds, showed a delayed but pronounced upregulation around day 15, consistent with a model of degradation in two‑phases, in which intracellular monooxygenation (P450s) produces soluble intermediates subsequently depolymerized by extracellular ligninolytic enzymes (Asemoloye et al. 2018; Ribeiro Tomé et al. 2024). Other studies in oil‑impacted soil have shown a similar behavior (Vaksmaa et al. 2020), supporting our observation of a mid‑treatment fungal laccase peak. The constant expression of *tilA* is consistent with the key role that laccase plays in *Aspergillus* adaptation to toxic environmental conditions such as contamination with crude oil (Alharbi et al. 2022). The delay observed in the overexpression of *tilA* and *dyp* could be related to an initial adaptation phase required for *A. oryzae* and *P. citrinum* to modify their metabolism in response to asphaltenes and to deploy more specific catabolic mechanisms for its degradation after the first week of treatment, similar to how the adaptation of *A. oryzae* has been reported during pesticide degradation in soil-based media (Barberis et al. 2019). Further, the biosurfactant and emulsifying activities reported for *P. citrinum* possibly increased substrate accessibility and potentiated the synergistic action of intracellular P450s and extracellular laccases/DyPs, accelerating the co-metabolic degradation of the heavier fractions of petroleum (Costa et al. 2023). This could also indicate that other genes that encode oxidative extracellular enzymes, different from those evaluated in this study, could be induced during the first days of asphaltene degradation (e.g., other peroxidases, cellulases, or lipases) (Asemoloye et al. 2018) and that co-metabolism is likely involved in this process (Nzila and Musa 2021).

Overexpression of most of the genes evaluated on day 15 of asphaltene degradation reinforces the idea of a previous adaptation of the consortium to the contaminated soil, possibly contributing to sustained mineralization of asphaltenes. In contrast, the expression of the chloroperoxidase-encoding gene (*cpo*) was downregulated in the presence of asphaltenes and appeared to play a limited role in the degradation of asphaltenes by the fungal consortium. Although chloroperoxidase can catalyze petroporphyrin transformations and alter the aromaticity of asphaltenes under controlled conditions, previous findings indicate that its contribution to their biodegradation is context-dependent and often limited (Hernández-López et al. 2015). Cytochrome P450 and extracellular ligninolytic enzymes have been reported to play a larger role, which may better explain the *cpo* expression patterns observed in this study. The results revealed that genes encoding cytochrome P450 monooxygenases are the main players in the early stages of asphaltene degradation by the fungal consortium, followed by the simultaneous expression of genes encoding extracellular ligninolytic genes (laccase and DyP-peroxidase), dioxygenases, and monooxygenases, of which the latter two are overexpressed until the late stages of the process. These findings suggest that the metabolic mechanisms of both *A. oryzae* and *P. citrinum* are activated at different stages of degradation, but could contribute together synergistically to the efficient mineralization of asphaltenes.

## Conclusion

In this study, a novel tolerant, and highly efficient fungal consortium was developed for the biodegradation of asphaltenes. This robust, non-antagonistic fungal consortium can use heavy crude oil as the sole carbon source, tolerate remarkably high asphaltene levels, and efficiently degrade asphaltenes in contaminated soils. This research contributed to the identification of specific metabolic expression profiles involved in the mineralization of asphaltenes by filamentous fungi, consisting of the sequential expression of cytochrome P450 monooxygenases, extradiol dioxygenases, laccases, and DyP-peroxidases of *Aspergillus oryzae* and *Penicillium citrinum*. This metabolic response confers adaptive advantages to the consortium that improves its survival and effective mineralization of asphaltenes in heavily contaminated soils. The findings of this study provide new insights into the mechanisms of asphaltene degradation by filamentous fungi and confirm the ability of this fungal consortium, along with a biostimulation strategy, to be applied in soil bioremediation.

## Data Availability

The data supporting the findings of this study are available within the paper. Should any raw data files be needed in another format, they are available from the corresponding author upon reasonable request.

## References

[CR1] Alharbi NK, Alzaban MI, Albarakaty FM, Abd El-Aziz ARM, AlRokban AH, Mahmoud MA (2022) Transcriptome profiling reveals differential gene expression of laccase genes in *Aspergillus terreus* KC462061 during biodegradation of crude oil. Biology 11:564. 10.3390/biology11040564 35453763 10.3390/biology11040564 PMC9026905

[CR2] Al-Zaban MI, AlHarbi MA, Mahmoud MA (2021) Hydrocarbon biodegradation and transcriptome responses of cellulase, peroxidase, and laccase encoding genes inhabiting rhizospheric fungal isolates. Saudi J Biol Sci 28:2083–2090. 10.1016/J.SJBS.2021.01.009 33935563 10.1016/j.sjbs.2021.01.009 PMC8071968

[CR3] Asemoloye MD, Ahmad R, Jonathan SG (2018) Transcriptomic responses of catalase, peroxidase and laccase encoding genes and enzymatic activities of oil spill inhabiting rhizospheric fungal strains. Environ Pollut 235:55–64. 10.1016/J.ENVPOL.2017.12.042 29274538 10.1016/j.envpol.2017.12.042

[CR4] Asemoloye MD, Tosi S, Daccò C, Wang X, Xu S, Marchisio MA, Gao W, Jonathan SG, Pecoraro L (2020) Hydrocarbon degradation and enzyme activities of *Aspergillus oryzae* and *Mucor irregularis* isolated from Nigerian crude oil-polluted sites. Microorganisms 1912. 10.3390/microorganisms8121912 33266344 10.3390/microorganisms8121912 PMC7761101

[CR5] ASTM (2022) Standard test method for determination of asphaltenes (heptane insolubles) in crude petroleum and petroleum products. Annual Book of ASTM Standards, ASTM Standard D6560–22, West Conshohocken, PA. 10.1520/D6560-22

[CR6] Baker P, Tiroumalechetty A, Mohan R (2019) Fungal enzymes for bioremediation of xenobiotic compounds. In: Yadav A, Singh S, Mishra S, Gupta A (eds) Recent advancement in white biotechnology through fungi, volume 3: perspective for sustainable environments. Springer International Publishing 463–489. 10.1007/978-3-030-25506-0_19

[CR7] Barberis CL, Carranza CS, Magnoli K, Benito N, Magnoli CE (2019) Development and removal ability of non-toxigenic *Aspergillus* section Flavi in presence of atrazine, chlorpyrifos and endosulfan. Rev Argent Microbiol 51:3–11. 10.1016/j.ram.2018.03.002 29885942 10.1016/j.ram.2018.03.002

[CR8] Barnes NM, Khodse VB, Lotlikar NP, Meena RM, Damare SR (2018) Bioremediation potential of hydrocarbon-utilizing fungi from select marine niches of India. 3 Biotech 8:21. 10.1007/s13205-017-1043-8 10.1007/s13205-017-1043-8 PMC5735040 29276659

[CR9] Benguenab A, Chibani A (2021) Biodegradation of petroleum hydrocarbons by filamentous fungi (*Aspergillus ustus* and *Purpureocillium lilacinum*) isolated from used engine oil contaminated soil. Acta Ecol Sin 41:416–423. 10.1016/j.chnaes.2020.10.008

[CR10] Brown DM, Bonte M, Gill R, Dawick J, Boogaard PJ (2017) Heavy hydrocarbon fate and transport in the environment. Q J Eng Geol Hydrogeol 50:333–346. 10.1144/qjegh2016-142

[CR11] Buarque FS, Sales JCS, Lobo LC, Chrisman ECAN, Ribeiro BD, Coelho MAZ (2025) Asphaltenes biodegradation from heavy crude oils by the yeast *Yarrowia lipolytica*. Bioprocess Biosyst Eng 48:381–394. 10.1007/S00449-024-03114-0 39648210 10.1007/s00449-024-03114-0

[CR12] Cáceres-Zambrano JZ, Rodríguez-Córdova LA, Sáez-Navarrete CA, Rives YC (2024) Biodegradation capabilities of filamentous fungi in high-concentration heavy crude oil environments. Arch Microbiol 206:3. 10.1007/S00203-024-03835-6 10.1007/s00203-024-03835-6 38407586

[CR13] Costa ER, Souza AF, Takaki GM, Andrade RF (2023) Bioemulsifier production by *Penicillium Citrinum* UCP 1183 and microstructural characterization of emulsion droplets. In: Connecting Expertise Multidisciplinary Development for the Future. Seven Editora, Brazil, pp 1-10. 10.56238/Connexpemultidisdevolpfut-168

[CR14] Dinakarkumar Y, Ramakrishnan G, Gujjula KR, Vasu V, Balamurugan P, Murali G (2024) Fungal bioremediation: an overview of the mechanisms, applications and future perspectives. J Environ Chem Ecotoxicol 6:293–302. 10.1016/J.ENCECO.2024.07.002

[CR15] Essabri AM, Aydinlik NP, Williams NE (2019) Bioaugmentation and biostimulation of total petroleum hydrocarbon degradation in a petroleum-contaminated soil with fungi isolated from olive oil effluent. Water Air Soil Pollut 230:76. 10.1007/s11270-019-4127-8

[CR16] Fallahi M, Sarempour M, Mirzadi Gohari A (2023) Potential biodegradation of polycyclic aromatic hydrocarbons (PAHs) and petroleum hydrocarbons by indigenous fungi recovered from crude oil-contaminated soil in Iran. Sci Rep 13:1. 10.1038/s41598-023-49630-z 38092846 10.1038/s41598-023-49630-z PMC10719355

[CR17] Galitskaya P, Biktasheva L, Blagodatsky SS (2021) Response of bacterial and fungal communities to high petroleum pollution in different soils. Sci Rep 11:164. 10.1038/s41598-020-80631-4 33420266 10.1038/s41598-020-80631-4 PMC7794381

[CR18] Guirado M, García-Delgado C, Pindado O, de la Ortiz Torre B, Escolano O, Eymar E, Millán R (2023) Bioremediation study of a hydrocarbon-contaminated soil by profiling aromatic and aliphatic chains. Appl Soil Ecol 190:104983. 10.1016/j.apsoil.2023.104983

[CR19] Hall TA (1999) Bioedit: a user-friendly biological sequence alignment editor and analysis program for Windows 95/98/NT. Nucleic Acids Symp Ser 41:95–98

[CR20] Harlington AC, Shearwin KE, Bell SG, Whelan F (2022) Efficient O-demethylation of lignin monoaromatics using the peroxygenase activity of cytochrome P450 enzymes. Chem Commun 58:13321–13324. 10.1039/d2cc04698a 10.1039/d2cc04698a 36346098

[CR21] Harms H, Schlosser D, Wick LY (2011) Untapped potential: exploiting fungi in bioremediation of hazardous chemicals. Nat Rev Microbiol 9:177–192. 10.1038/nrmicro2519 21297669 10.1038/nrmicro2519

[CR22] Hassanzadeh M, Abdouss M (2023) Molecular structure: the first and most significant factor in the precipitation of asphaltenes. SPE J 28:894–907. 10.2118/212311-PA

[CR23] Hernández-López EL, Ayala M, Vazquez-Duhalt R (2015) Microbial and enzymatic biotransformations of asphaltenes. Petrol Sci Technol 33:1017–1029. 10.1080/10916466.2015.1014960

[CR24] Khan MF, Murphy CD (2022) Cytochrome P450 5208A3 is a promiscuous xenobiotic biotransforming enzyme in Cunninghamella elegans. Enzyme Microb Technol 161:110102. 10.1016/j.enzmictec.2022.110102 35917624 10.1016/j.enzmictec.2022.110102

[CR25] Khandelwal A, Singh SB, Sharma A, Nain L, Varghese E, Singh N (2023) Effect of surfactant on degradation of Aspergillus sp. and Trichoderma sp. mediated crude oil. Int J Environ Anal Chem 103:1667–1680. 10.1080/03067319.2021.1879800

[CR26] Kshirsagar SD, Mattam AJ, Jose S, Ramachandrarao B, Velankar HR (2020) Heavy hydrocarbons as selective substrates for isolation of asphaltene degraders: a substrate-based bacterial isolation strategy for petroleum hydrocarbon biodegradation. Environ Technol Innov 19:100832. 10.1016/J.ETI.2020.100832

[CR27] Kumar A, Chandra R (2020) Ligninolytic enzymes and its mechanisms for degradation of lignocellulosic waste in environment. Heliyon 19:3–170. 10.1016/j.heliyon.2020.e03170 10.1016/j.heliyon.2020.e03170 PMC7033530 32095645

[CR28] Kumar V, Kumar H, Vishal V, Lal S (2023) Studies on the morphology, phylogeny, and bioremediation potential of *Penicillium citrinum* and *Paecilomyces variotii* (Eurotiales) from oil-contaminated areas. Arch Microbiol 205:50. 10.1007/s00203-022-03383-x 36598589 10.1007/s00203-022-03383-x

[CR29] Li T, Jiang G, Qu H, Wang Y, Xiong Y, Jian Q, Wu Y, Duan X, Zhu X, Hu W, Wang J, Gong L, Jiang Y (2017) Comparative transcriptome analysis of Penicillium citrinum cultured with different carbon sources identifies genes involved in Citrinin Biosynthesis. Toxins 9:69. 10.3390/toxins9020069 28230802 10.3390/toxins9020069 PMC5331448

[CR30] Livak KJ, Schmittgen TD (2001) Analysis of relative gene expression data using real-time quantitative PCR and the 2(-Delta Delta C(T)) method. Methods 25:402–408. 10.1006/meth.2001.1262 11846609 10.1006/meth.2001.1262

[CR31] Mahmud T, Alhaji S, Ibrahim L, Zakari N, Danlami D, Shehu A (2022) Hydrocarbon degradation potentials of fungi: a review. J Environ Bioremed Toxicol 5:50–56. 10.54987/jebat.v5i1.681

[CR32] Navas-Cáceres OD, Parada M, Zafra G (2023) Development of a highly tolerant bacterial consortium for asphaltene biodegradation in soils. Environ Sci Pollut Res Int 30:123439–123451. 10.1007/s11356-023-30682-7 37982951 10.1007/s11356-023-30682-7 PMC10746765

[CR33] Ningombam L, Mana T, Pradhan S, Apum G, Singh YD (2025) Fungal bioremediation in environmental pollution and recent strategies. Discover Environment 3:100. 10.1007/S44274-025-00267-X

[CR34] Nzila A, Musa MM (2021) Current knowledge and future challenges on bacterial degradation of the highly complex petroleum products asphaltenes and resins. Front Environ Sci. 10.3389/fenvs.2021.779644

[CR35] Pavel AB, Vasile CI (2012) PyElph - a software tool for gel images analysis and phylogenetics. BMC Bioinformatics 13:9. 10.1186/1471-2105-13-9 22244131 10.1186/1471-2105-13-9 PMC3299638

[CR36] Ramoutar S, Mohammed A, Ramsubhag A (2019) Laboratory-scale bioremediation potential of single and consortia fungal isolates from two natural hydrocarbon seepages in Trinidad, West Indies. Bioremed J 23:131–141. 10.1080/10889868.2019.1640181

[CR37] Ribeiro Tomé LM, Dornelles Parise MT, Parise D, de Carvalho Azevedo VA, Brenig B, Badotti F, Góes-Neto A (2024) Pure lignin induces overexpression of cytochrome P450 (CYP) encoding genes and brings insights into the lignocellulose depolymerization by *Trametes villosa*. Heliyon 10:e28449. 10.1016/j.heliyon.2024.e28449 38689961 10.1016/j.heliyon.2024.e28449 PMC11059554

[CR38] Shahebrahimi Y, Fazlali A, Motamedi H, Kord S, Mohammadi AH (2020) Effect of various isolated microbial consortiums on the biodegradation process of precipitated asphaltenes from crude oil. ACS Omega 5:3131–3143. 10.1021/acsomega.9b02056 32118129 10.1021/acsomega.9b02056 PMC7045313

[CR39] Simmons K (2023) Operating procedure: Soil Sampling. ID: LSASDPROC-300-R5. U.S. Environmental Protection Agency. https://www.epa.gov/sites/default/files/2015-06/documents/Soil-Sampling.pdf. Accessed 16 Jan 2026

[CR40] Tamano K, Bruno KS, Karagiosis SA, Culley DE, Deng S, Collett JR, Umemura M, Koike H, Baker SE, Machida M (2013) Increased production of fatty acids and triglycerides in *Aspergillus oryzae* by enhancing expressions of fatty acid synthesis-related genes. Appl Microbiol Biotechnol 97:269. 10.1007/s00253-012-4193-y 22733113 10.1007/s00253-012-4193-y

[CR41] Tamura K, Stecher G, Kumar S (2021) MEGA 11: molecular evolutionary genetics analysis version 11. Mol Biol Evol 38:3022–3027. 10.1093/molbev/msab120 33892491 10.1093/molbev/msab120 PMC8233496

[CR42] Vaksmaa A, Guerrero Cruz S, Ghosh P, Zeghal E, Hernando-Morales V, Niemann H (2023) Role of fungi in bioremediation of emerging pollutants. Front Mar Sci. 10.3389/fmars.2023.1070905

[CR43] White TJ, Bruns TD, Lee SH, Taylor J (1990) Amplification and direct sequencing of fungal ribosomal RNA genes for phylogenetics, In: Innis M, Gelfand D, Sninsky J, White TJ (eds) PCR protocols: a guide to methods and applications. Academic Press 315–322. 10.1016/B978-0-12-372180-8.50042-1

[CR44] Yanto DHY, Hidayat A (2020) Biodegradation of buried crude oil in soil microcosm by fungal co-culture. IOP Conf Ser: Mater Sci Eng 980:012084. 10.1088/1757-899X/980/1/012084

[CR45] Yanto DHY, Tachibana S (2014) Enhanced biodegradation of asphalt in the presence of Tween surfactants, Mn2+ and H2O2 by *Pestalotiopsis* sp. in liquid medium and soil. Chemosphere 103:105–113. 10.1016/j.chemosphere.2013.11.044 24331036 10.1016/j.chemosphere.2013.11.044

[CR46] Yanto DHY, Tachibana S (2014) Potential of fungal co-culturing for accelerated biodegradation of petroleum hydrocarbons in soil. J Hazard Mater 278:454–463. 10.1016/j.jhazmat.2014b.06.039 24997261 10.1016/j.jhazmat.2014.06.039

[CR47] Zafra G, Absalón ÁE, Cuevas MDC, Cortés-Espinosa DV (2014) Isolation and selection of a highly tolerant microbial consortium with potential for PAH biodegradation from heavy crude oil-contaminated soils. Water Air Soil Pollut 225:1826. 10.1007/s11270-013-1826-4

[CR48] Zafra G, Taylor TD, Absalón AE, Zafra G, Absalón Á, Anducho-Reyes M, Fernandez FJ, Cortés-Espinosa DV (2017) Construction of PAH-degrading mixed microbial consortia by induced selection in soil. Chemosphere 172:120–126. 10.1016/j.chemosphere.2016.12.038 28063314 10.1016/j.chemosphere.2016.12.038

[CR49] Zhang R, Sun S, Wang L, Li G, Shi Q, Jia J, Zhang X, Yu H, Xie S (2021) Lignin structure defines the properties of asphalt binder as a modifier. Constr Build Mater 310:125156. 10.1016/j.conbuildmat.2021.125156

[CR50] Zhu H, Ren L, Yang H, Zhang J (2025) Leveraging indigenous Bacillus consortia for heavy oil biodegradation and soil bioremediation. Environmental Technology & Innovation 40:104415. 10.1016/J.ETI.2025.104415

